# The Contribution of Digital Health in the Response to Covid-19 in Vietnam

**DOI:** 10.3389/fpubh.2021.672732

**Published:** 2021-08-24

**Authors:** Long Viet Bui, Son Thai Ha, Ha Ngoc Nguyen, Truong Thanh Nguyen, Thuy Phuong Nguyen, Kien Tran, Tuyen Van Tran, Tu Huu Nguyen, Thong Huy Tran, Nghiem Duc Pham, Hanh My Bui

**Affiliations:** ^1^Centre for Research, Consulting and Support of Community Health, Hanoi, Vietnam; ^2^Administration of Medical Services – Ministry of Health, Hanoi, Vietnam; ^3^People's Police Academy, Hanoi, Vietnam; ^4^Ministry of Science and Technology, Hanoi, Vietnam; ^5^Menzies Institute for Medical Research, University of Tasmania, Hobart, TAS, Australia; ^6^School of Law, Vietnam National University, Hanoi, Vietnam; ^7^eHealth Administration – Ministry of Health, Hanoi, Vietnam; ^8^Vietnam Young Physician Associations, Hanoi, Vietnam; ^9^Vietnam Climate Innovation Center, Hanoi, Vietnam; ^10^Department of Tuberculosis and Lung Disease, Hanoi Medical University, Hanoi, Vietnam; ^11^Department of Functional Exploratory, Hanoi Medical University Hospital, Hanoi, Vietnam

**Keywords:** digital health, COVID-19, public health, policy, Vietnam

## Abstract

Emerging from early of 2020, the COVID-19 pandemic has become one of the most serious health crisis globally. In response to such threat, a wide range of digital health applications has been deployed in Vietnam to strengthen surveillance, risk communication, diagnosis, and treatment of COVID-19. Digital health has brought enormous benefits to the fight against COVID-19, however, numerous constrains in digital health application remain. Lack of strong governance of digital health development and deployment; insufficient infrastructure and staff capacity for digital health application are among the main drawbacks. Despite several outstanding problems, digital health is expected to contribute to reducing the spread, improving the effectiveness of pandemic control, and adding to the dramatic transformation of the health system the post-COVID era.

## Introduction

The COVID-19 pandemic has created devastating social, economic and political impacts, challenging governments to act promptly to control the pandemic (1). Despite numerous constraints, Vietnam has been lauded for vigorous efforts in fighting the pandemic, including strong political commitment and whole-society response approach such as intensive surveillance, contact tracing, case management, massive quarantine and lock down, and transparent risk communication (2). Contributing to such success was the role of Digital Health (DH), which has not been discussed systematically elsewhere.

DH is defined as “the use of information and communications technology in support of health and health-related fields” (3), including but not limited to mobile mHealth, health information technology, wearable devices, telehealth and telemedicine, artificial intelligence (AI) and personalised medicine (4). DH plays an important role in improving effectiveness of the health system, increasing accessibility to health services while containing healthcare cost, and in working toward universal health coverage (3). DH demonstrated its prospects in infectious diseases control (5, 6). DH technology has been deployed to address the most urgent needs during the COVID-19 pandemic, including immediate outbreak response and later, impact mitigation (7). Evidence from prior research suggests that adoption of digital health, especially telemedicine and virtual care was considerably increasing during the COVID-19 and offer considerable benefits to the health care as weel ass society (8–11).

Vietnam has embraced the digital technologies in healthcare early, such as telemedicine, hospital information system, mHealth quite early (12–14). Amid rapid spreading of the epidemic around the world, there were several initiatives to implement DH applications in response to the COVID-19 pandemic in Vietnam. In this article, we aimed to provide an overview on the implementation of DH technologies the fight against COVID-19 in Vietnam.

## Methods and Materials

We examined the use of digital health in Vietnam in accordance with the epidemiological development of COVID-19 in Vietnam. Although there have been several articles discussing the COVID-19 prevention experience in Vietnam (2, 15–18), there is lack of peer-reviews articles that focus on DH. We collected and summarised data and information on the use of DH from the official portal of the Ministry of Health (MOH) in Vietnam on the COVID-19 pandemic and existing legal documents, official reports of the Ministry of Health from Jan 2020 to Feb 2021. For additional information, we conducted a search on Google with keywords related to COVID-19 including “telehealth,” “telemedicine,” “digital health” “e-health,” “m-health,” “COVID-19,” “coronavirus 2019 and “ncov.” The data was entered in a standardised spreadsheet. Any discrepancies in data extraction were discussed between the researchers to reach consensus. If it cannot be resolved, a third researcher was consulted. We also employed the same epidemiological data which was used in previous study (19).

## Results

**Figure 1:**
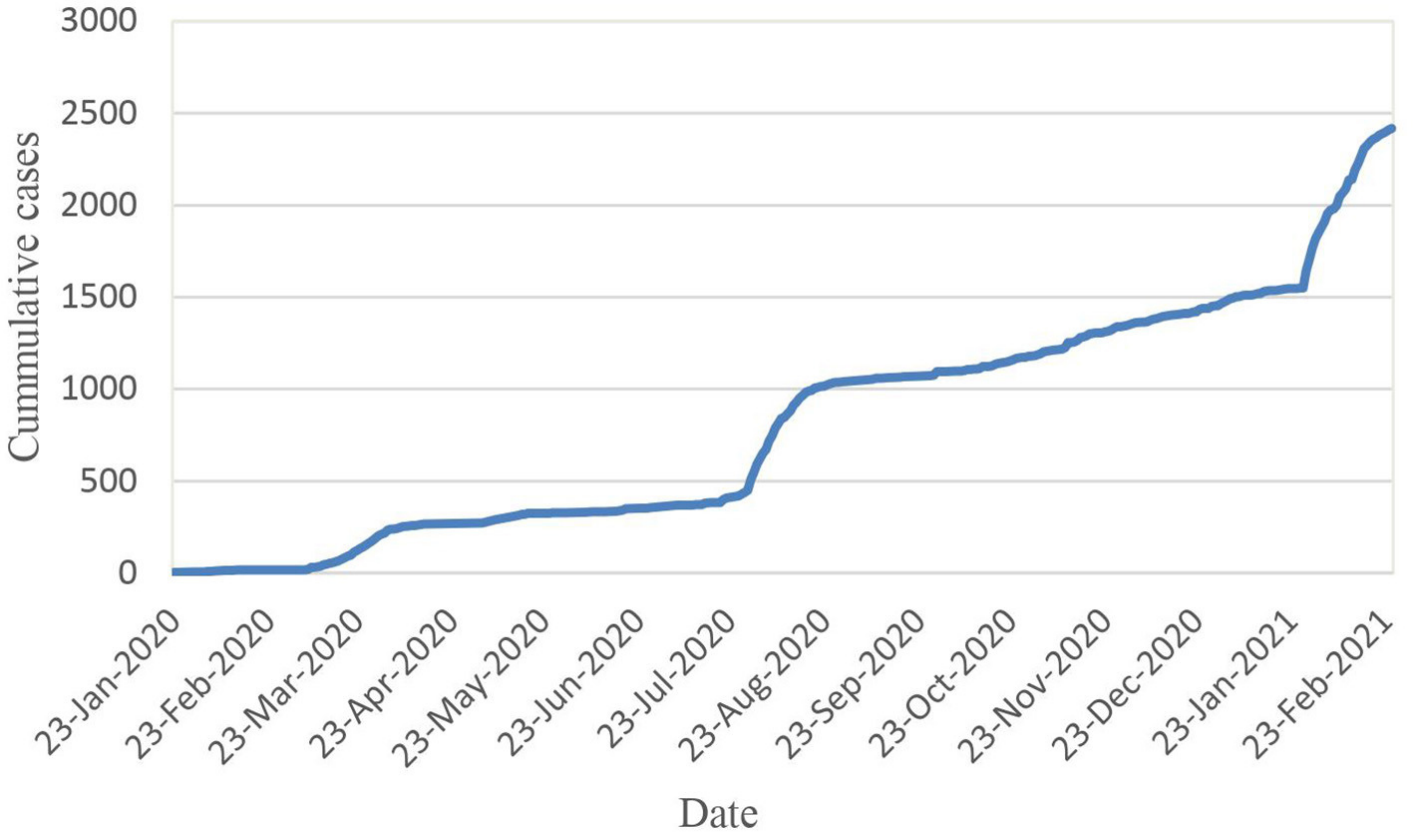
Cumulative COVID-19 cases recorded in Vietnam from 23 Jan 2020 to 23 Feb 2021.

There are 07 major DH applications were implemented in Vietnam since the very beginning of COVID-19 pandemic. They were categorised into four groups based on their main purpose, including surveillance and contact tracing, health communication, telemedicine and Artificial Intelligence to support diagnosis and treatment. Table 1 summarised the roll out of DH applications, in according with the epidemiological curve of COVID-19 of Vietnam from 23 Feb 2020 to until 26 Feb 2021 (Figure 1).

**Table 1:** List of digital Health applications in addressing COVID-19 in Vietnam.

**No**	**Digital health interventions**	**Launching** **date**	**Main purpose**
1	Electronic communicable disease surveillance software	2004	Surveillance
2	https://ncov.moh.gov.vn	8-Feb-20	Communication
3	Vietnam Health app	8-Feb-20	Communication
4	Virtual assistant (chatbot) on COVID-19	14-Feb-20	Communication
5	Bluezone app	18-Feb-20	Contact tracing
6	NCOVI, Vietnam health declaration apps	9-Mar-20	Health declaration
7	COVID-19 case management software	26-Mar-20	Surveillance, case management
8	Telemedicine Center for COVID-19 Outbreak Control	5-Mar-20	Telemedicine
9	Pilot program on telemedicine	18-Apr-20	Telemedicine
10	DrAid software	25-Aug-20	Diagnosis and treatment support

### Surveillance and Contact Tracing

The national electronic communicable disease surveillance system (eCDS), developed in 2004, was the first nationwide attempt to computerised surveillance data. eCDS mainly focuses primarily on reporting of case-based hospital admissions through an electronic system – the eCDS software. Several diseases-specific or syndrome-specific sentinel surveillance programs complement eCDS system, focusing on selected conditions such as Dengue; Hand, Foot, and Mouth Disease; Japanese Encephalitis virus; influenza-like illness; severe acute respiratory infections; and Zika (20). When the first COVID-19 case was reported (21), the Government of Vietnam considered COVID-19 as a Group A (extremely dangerous) communicable disease and needed to be included immediately in the eCDS accordingly to Law on prevention and control of infectious diseases (22). However, the eCDS only captures administrative information, epidemiological history, date of detection and confirmation, date of discharge, and discharge status of suspected and confirmed cases. Follow-up clinical information are unavailable. Given this problem, the COVID-19 online case management software (CMS) was developed by the Administration of Medical Services (AMS), Vietnam Ministry of Health (MOH). In addition to administrative information and epidemiological history, the health facilities are required to routinely input clinical manifestations, laboratory and image diagnosing results of patients. In complement with the eCDS, the CMS is helpful on providing detailed information on COVID-19 situation for prompt control measures and risk communication.

### Health Communication

The information on policy, control measures and chronology of COVID-19 patients are daily publishing on the official portal on COVID-19 of the MOH https://ncov.moh.gov.vn, which was launched on 08 February 2020. Many newspapers have used information from this portal to publish update news, contributing to a large number of communications to raise public awareness about the disease and inform the community on disease prevention and protection strategies (23). Notably, several research on COVID-19 in Vietnam relied from daily communication on the official portal as well as other newspaper to exact data for analysis (15, 16, 19). On the same day, a smartphone app called “Sú'c Khçe Việt Nam” (Việt Nam Health) was introduced, providing updated epidemic information and self-assessment guides for COVID-19 infection. If there is a risk of infection, users can send information to health facilities in their living areas for instructions on self-isolation.

In addition, two mobile apps (NCOVI and Vietnam Health Declaration) were developed to record electronic health declaration form for domestic and international travellers for the purpose of case monitoring and surveillance (24). People can also access two websites https://suckhoetoandan.vn/khaiyte and https://tokhaiyte.vn and follow the instructions to perform the steps of electronic medical declaration. Bluezone, a Bluetooth-based app, was rolled out on 18 April 2020 to link smartphones that come within 6 feet of each other and record the time and duration of contact. When a user of Bluezone became a confirmed COVID-19 case, health authorities will enter the patient's information into the system. The app then identifies the case' contact history during the 14-day period from onset date and notifies these users that they are at risk of infection and instructs them to contact health authorities (15). The data exacted from Bluezone and health declaration system play important roles in contact tracing. Although there are several concerns on the privacy of the app (25), the developers claim that the app does not collect data on users' locations and that the collected information is anonymous. Only authorised health authorities could be able to access to the identities of infected people and those who came into close contact with them for the purpose of contact tracing. The government highly encourages residents to download and use the app. Installing and using the Bluezone is a requirement to be exempted from centralised quarantine for short-term visitors to the country (26).

### Telemedicine

The pandemic has created many challenges in delivery of health care services. Redirecting resources in the health sector to combat the pandemic has put a strain on many other health services. On the other hand, aggressive control measures such as social distancing, city lockdown and hospital closure have seriously affected people's access to health services (10). As a country suffering a double burden of disease, of which non-communicable diseases occupy 10 positions out of the 12 biggest causes of death, the pressure on the health system to provide services in the context of COVID-19 pandemic is getting more challenging (27). At the same time, many people with symptoms of respiratory illness put great pressure on COVID-19 testing and screening. These challenges have forced the health sector to change to maintain the delivery of services. Telemedicine is one of the solutions to provide medical services without traditional face-to-face consultations between physicians and customer (28).

On 5 Mar 2020, The AMS launched Telemedicine Centre for COVID-19 Outbreak Control. The centre is responsible for managing resources and providing technical support for health clinics in admitting, quarantining, diagnosing, and treating COVID-19 patients indirectly or virtually, especially for clinics in remote areas (18). The Center has contributed greatly to the very low COVID mortality rate in Vietnam (17).

On 16 April 2020, the MOH has announced a telemedicine program aiming to promote virtual medical examination and treatment to mitigate the risk of COVID-19, to foster digital transformation in the health sector, and to improve the quality of healthcare for people. The pilot program was launched on 18 April 2020 at the Hanoi Medical University Hospital and several satellite hospitals. On 22 June 2020, the project “Remote health examination and treatment from 2020 to 2025” has been approved by the Ministry of Health. After a 2-month piloting, on 25 September 2020, the project has successfully connected 1,000 health facilities and clinics in remote mountainous regions with 30 tertiary hospitals in Hanoi and Ho Chi Minh City to provide remote consultation and treatment (29).

### Artificial Intelligence to Support Diagnosis and Treatment

On February 14, 2020, the eHealth Administration officially launched a virtual assistant (chatbot) on COVID-19 (30). The virtual assistant is developed based on artificial intelligence technologies, capable of automatic, continuous real-time answers that allow multiple users to ask and answer questions on COVID-19 related information simultaneously.

On 25 August 2020, Vingroup offered the MOH a software named DrAid and attached devices to assist with prognostic evaluation in the treatment of COVID-19. Using Virtual Label Generative Adversarial Networks, DrAid is able to classify anomalies in <5 s based on upright chest X-ray images. The software is expected to boost the accuracy of the test results, in combination with the PCR test (31).

## Discussion

Like other countries, Vietnam has seen a plethora of DH applications during the COVID-19 (32). Despite the rapid deployment of applications, including Telemedicine and AI, DH application in Vietnam is still facing numerous constrains. In terms of institutional perspective, the DH applications for combating COVID-19 are not centrally developed and managed. They have been developed in a very short time and administered by many different agencies. It has reflected the inflexible and overlapping of health authorities in the structure of Vietnamese health system (33). The discretion of applications, such as eCDS, CMS, has highlighted the need of standardised and centralised data warehouse for healthcare in Vietnam, which was initiated in 1995 (33, 34). The absence of a centralised database hinders the potential power of cutting-edge technologies in automation of big data mining, analytical, visualisation and modelling, and artificial intelligence to support diagnosing, managing and triaging (35). Although eCDS and CMS software have satisfied the information needs in fighting the pandemic, they are largely dependent on health workers to input data into a computer-based spreadsheet, confining heath authorities to achieve real-time manner of the surveillance system as patient volume increased. It also lacks consistent system in place to notify a suspected case or to track interfacility transfer of confirmed COVID-19 patient as very few hospitals in Vietnam have standard Electronic Medical Record (EMR) despite 100% of hospitals have implemented hospital information systems (36). When combined with EHR and dedicated devices, such as wearables (37), telemedicine provides additional benefits of enhancing patient-provider connectivity, creating innovative model of care, allowing physicians to view patient records remotely, prescription, lab reports, not just merely virtual communication (38). Regulations on medical procedures and protocols at the national level for telemedicine to be optimised are unavailable (29).

The utilisation of DH in the health system faces several challenges such as insufficient financial resources to invest in infrastructure, the disparity in information technology literacy of physicians and health workers at all levels. Moreover, there has not been standardised guidance for digital health services delivery for local health staff while the application of DH at local level has been scaling up too fast. That could put an extra burden on the health system as the COVID-19 pandemic continues. Additionally, all COVID-19 response activities are centrally managed by the government, not all activity data have been standardised and digitalized into a national database on COVID-19 to be used by DH applications to fight this pandemic. It is high time to conduct a health technology assessment to quantifying the effectiveness of electronic medical applications to address COVID-19 to solving outstanding institutional and technical problems, as well as figure out determinants that affecting the adoption of digital health (9). More importantly, this is an opportunity to help create a sound view and expectation of the public on DH technologies (33), removing barriers and restrictions on the provider's perspective, such as fear of medical errors, and toward consumer's perspective, concerning on privacy, confidentiality, and security enhancement (29).

The National Digital Transformation Programme toward 2025 with a vision to 2030 has been approved on 22 December 2020 (39), with specific tasks and solutions in developing digital transformation platforms in the health sector, including but not limited to legislature and institutional building, digital infrastructure development, digital data development, ensuring security, human resource development. The success of DH in the COVID-19 era may provide rewarding experience for the implementation of this programme.

Our article has several strengths, including being the first, to our best knowledge, to review the application of DH in the fight against COVID-19 in Vietnam in accordance with the epidemiological development of the pandemic. Our search captured a wide range of published and grey literature on the use of DH for COVID-19 control. However, there were potential limitations in our study. We could not quantify the contribution of DH in the response to COVID-19 due to lack of sufficient purpose-built evaluation of DH application as well as the readiness of the stakeholders in using these DH applications during the pandemic. This has yielded the need to systematically assess the effectiveness of DH in disease control and COVID-19 response.

## Conclusions

This article provided a review on the application of DH technology in responding to COVID-19 in Vietnam. Despite several outstanding problems, DH is expected to contribute to reducing the spread, improving the effectiveness of pandemic control, and contributing to the dramatic transformation of the post-COVID era. To optimise the application of DH, we need more evidence on the effectiveness of DH applications as well as facilitators and barriers in applying DH. This would be the basis to develop comprehensive policies to facilitate the advancement of DH in the future.

## Data Availability Statement

The original contributions presented in the study are included in the article/supplementary material, further inquiries can be directed to the corresponding author/s.

## Author Contributions

HMB, LVB, STH, and HNN: conceptualization. HMB, LVB, STH, and TTN: methodology. LVB, HMB, THN, STH, THT, and KT: writing—original draft. LVB, STH, HNN, TTN, THN, KT, TVT, THT, NDP, PTN, and HMB: writing—review and editing. All authors contributed to the article and approved the submitted version.

## Conflict of Interest

The authors declare that the research was conducted in the absence of any commercial or financial relationships that could be construed as a potential conflict of interest.

## Publisher's Note

All claims expressed in this article are solely those of the authors and do not necessarily represent those of their affiliated organizations, or those of the publisher, the editors and the reviewers. Any product that may be evaluated in this article, or claim that may be made by its manufacturer, is not guaranteed or endorsed by the publisher.
